# The effect of particle shape on discharge and clogging

**DOI:** 10.1038/s41598-021-82744-w

**Published:** 2021-02-08

**Authors:** Ahmed Hafez, Qi Liu, Thomas Finkbeiner, Raed A. Alouhali, Timothy E. Moellendick, J. Carlos Santamarina

**Affiliations:** 1 Earth Science and Engineering, KAUST, Thuwal, 23955-6900 Saudi Arabia; 2 Saudi Aramco, Dhahran, 31 311 Saudi Arabia

**Keywords:** Energy science and technology, Engineering, Physics

## Abstract

Granular flow is common across different fields from energy resource recovery and mineral processing to grain transport and traffic flow. Migrating particles may jam and form arches that span constrictions and hinder particle flow. Most studies have investigated the migration and clogging of spherical particles, however, natural particles are rarely spherical, but exhibit eccentricity, angularity and roughness. New experiments explore the discharge of cubes, 2D crosses, 3D crosses and spheres under dry conditions and during particle-laden fluid flow. Variables include orifice-to-particle size ratio and solidity. Cubes and 3D crosses are the most prone to clogging because of their ability to interlock or the development of face-to-face contacts that can resist torque and enhance bridging. Spheres arriving to the orifice must be correctly positioned to create stable bridges, while flat 2D crosses orient their longest axes in the direction of flowlines across the orifice and favor flow. Intermittent clogging causes kinetic retardation in particle-laden flow even in the absence of inertial effects; the gradual increase in the local particle solidity above the constriction enhances particle interactions and the probability of clogging. The discharge volume before clogging is a Poisson process for small orifice-to-particle size ratio; however, the clogging probability becomes history-dependent for non-spherical particles at large orifice-to-particle size ratio and high solidities, i.e., when particle–particle interactions and interlocking gain significance.

## Introduction

Granular flow is common across different fields including silo discharge in the food industry^1^, cement production^2^ and the transport of mining products^3^. Granular flow is often driven by fluids as in particle-laden fluid flow in porous media during water extraction^4^ and oil production^5–8^. In either case, migrating particles may jam and form arches that span constrictions and hinder particle flow^9^. Similar granular flow phenomena emerge in other fields, from vehicular transportation to crowd management at exit points^10^.

Many experimental, theoretical and numerical studies have investigated the migration and clogging of spherical particles^4,10–14^. Results highlight the central role of the constriction-to-particle size ratio *d*_*o*_/*d* in clogging, and show that the probability of clogging depends on the solidity *Φ* in the flowing fluid, defined as the ratio of the solid volume of migrating particles to the total fluid volume^15^.

Natural particles are rarely spherical, but exhibit eccentricity, angularity and roughness^1,16,17^. Shape emerges as a critical parameter in lost circulation treatment during oil drilling^5,18,19^, sand control methods^20,21^ and in the development of novel construction materials^22,23^. Previous research has investigated the role of particle shape on packing density and mechanical properties of granular media^24,25^. Experimental evidence and numerical simulation results show that particle interlocking governs the behavior of non-convex particles, while face-to-face aggregation leads to high packing densities in faceted particles^26,27^. Furthermore, ellipsoidal particles align their longest axes along the direction of flow^3,28^. The discharge rate of faceted and non-convex particles through a hopper during dry granular flow is lower than the flow rate of smooth spheres^1,22,29^.

This study evaluates the discharge of spherical, elongated, faceted, and non-convex particles during both dry-granular and particle-laden fluid flow. Experimental results and functions with physically justifiable asymptotic trends and with a minimum number of parameters (Ockham’s criterion) enable us to compare the clogging tendencies for different shapes.

## Experimental studies: materials and methods

**Figure 1:**
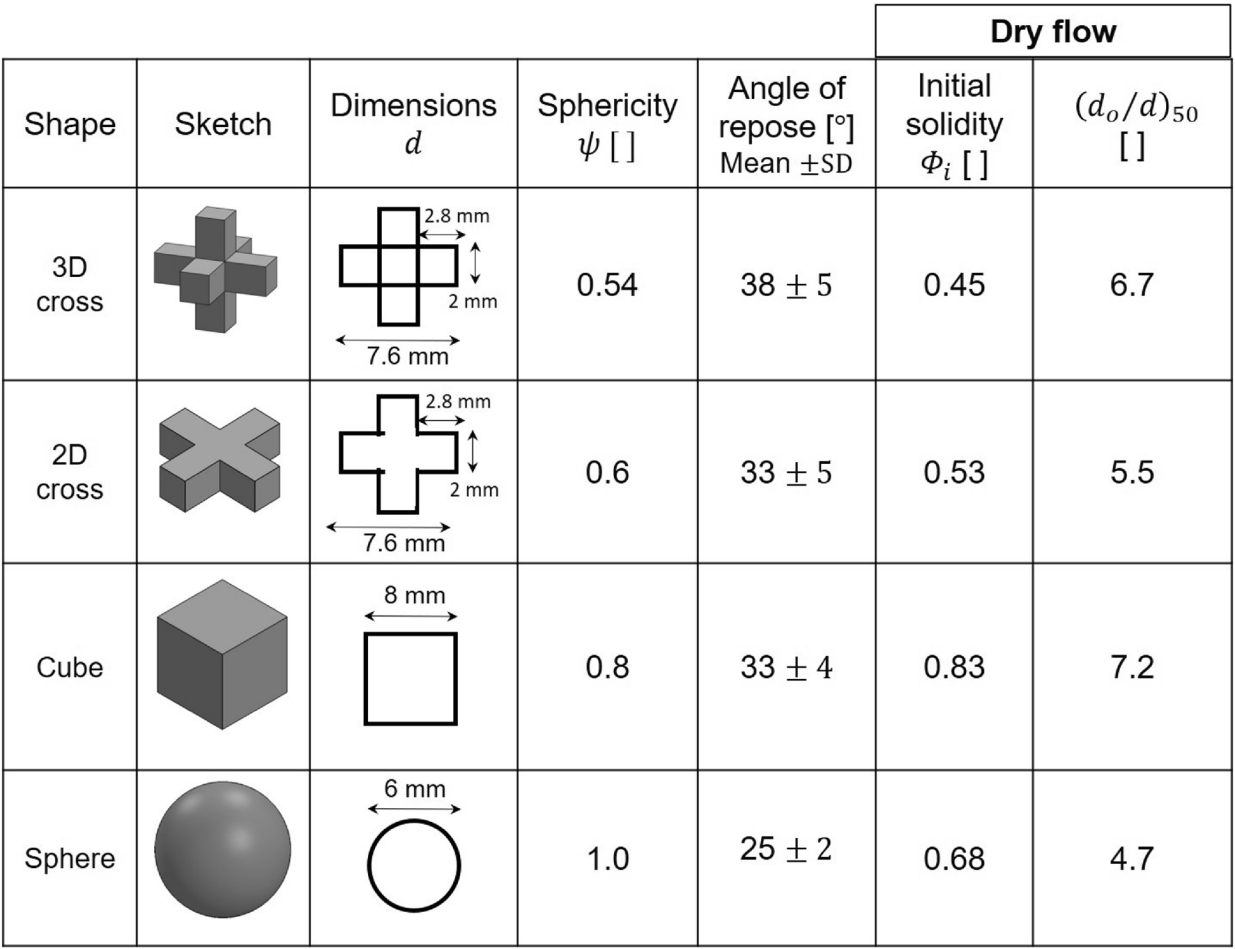
Particle shapes tested in this study. The angle of repose expressed as the mean ± the standard deviation SD. The initial packing density or solidity inside the hopper before discharge *Φ*_*i*_. The characteristic size ratio (*d*_*o*_/*d*)_*50*_ obtained by fitting Eq. (2) to data in Fig. 3.

Experiments involve plastic particles of four different shapes: cube, 2D cross, 3D cross and sphere (see Fig. 1 for dimensions). The 2D and 3D crosses were 3D printed with acrylonitrile butadiene styrene plastic filament (ABS: *ρ*_*p*_  = 1.04 g/cm^3^. Printer: Fortus 400 mc, Stratasys—STL files provided as supplementary materials). We purchased the plastic cubes (*ρ*_*p*_ = 1.09 g/cm^3^) and spheres (*ρ*_*p*_ = 1.02 g/cm^3^) and inspected them for consistency. The particle sphericity *ψ* relates the particle surface area ${A}_{p}$ to that of a sphere of the same volume ${V}_{p}$^17,30^:

1 $$\psi =\frac{\sqrt[3]{36\pi {V}_{p}^{2}}}{{A}_{p}}$$

Clearly, sphericity decreases as the particle shape deviates from a spherical geometry for which *ψ* = 1. Figure 1 lists the value of sphericity *ψ *for the particles tested in this study.

Particle shape determines mobility, interlocking and the angle of repose^31^. We measure the angle of repose on a rough surface to hinder particle rolling and follow these steps: (1) fill a cylinder with the selected particles, (2) slowly lift the cylinder to let the particles flow and form a granular heap, and (3) measure the angle the heap forms with the horizontal surface close to the base of the heap. We repeat the experiment for each shape five times and measure two diametrically opposite angles of repose. Results in Fig. 1 confirm the inverse relationship between sphericity and angle of repose.

**Figure 2:**
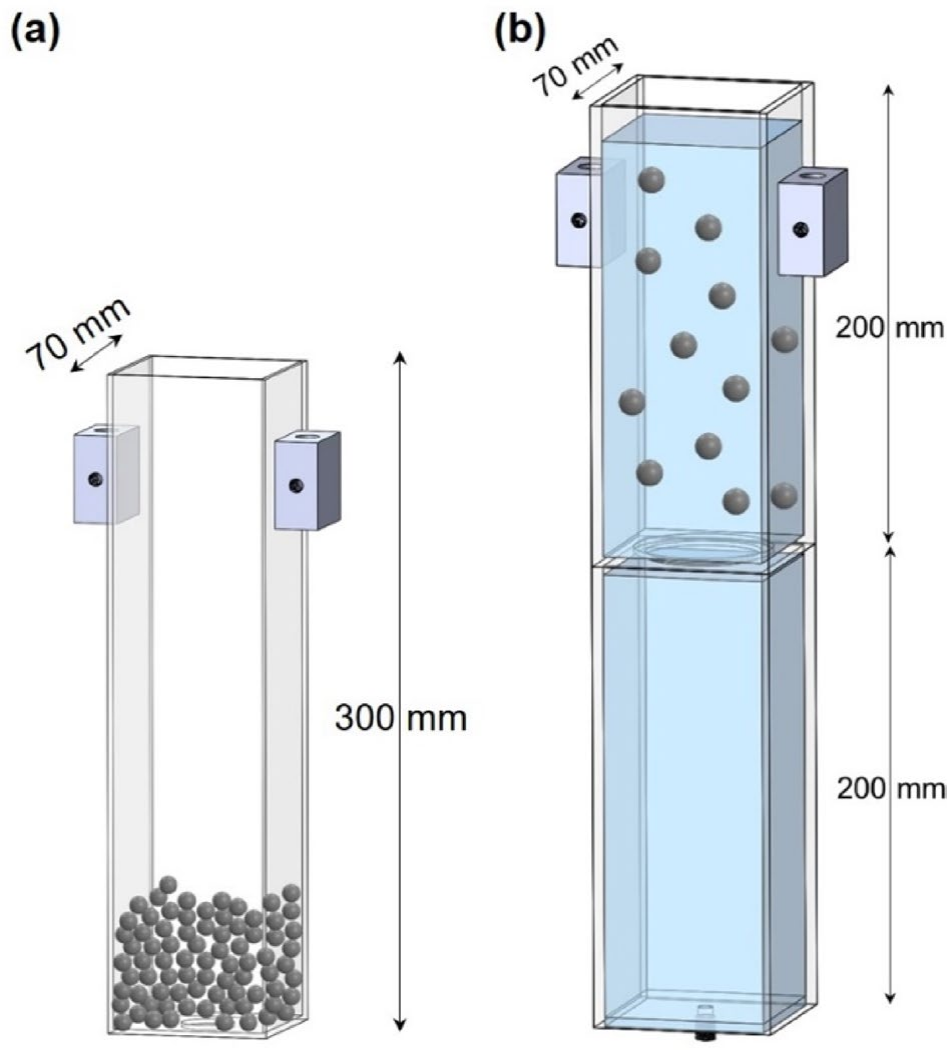
Hoppers. (**a**) Dry granular flow tests. (**b**) Particle-laden fluid flow tests; the dual chamber configuration allows for controlled discharge rate.

We use a flat-bottom hopper for dry granular flow experiments (Fig. 2a). All tests follow the same procedure: we close the orifice and fill the hopper with the selected particles (spheres, 2D cross and 3D cross: 4000 particles; only 2000 cubic particles fit inside the hopper due to the larger particle volume); then we uncover the orifice and let the particles flow until they form a bridge and clog the orifice, or the hopper is emptied. We run these experiments for different shapes and *d*_*o*_/*d* size ratios and repeat each test 20 times. The initial packing density or solidity *Φ*_*i*_ of particles inside the hopper before discharge varies for the different shapes (Fig. 1).

The device for particle-laden fluid flow involves the two-compartment hopper shown in Fig. 2b with an adjustable orifice size to accommodate *d*_*o*_/*d* ratios between 1.5 and 7.5; the double compartment design allows us flow rate control through the lower outlet regardless of the *d*_*o*_/*d* ratio. We use a viscous aqueous solution of xanthan gum to suspend particles (mass concentration of xanthan gum = 0.002, viscosity = 93 cp, mass density *ρ*_*f*_ = 0.99 g/cm^3^). The particle settling velocity determined from sedimentation tests is lower than 0.17 mm/s for all shapes while the initial particle discharge velocity exceeds 5 mm/s. Given the similarity in mass density *ρ*_*p*_/*ρ*_*f*_ = 1.05 and the low Stokes velocity, we assume that particles flow neutrally buoyant without significant deviations from the fluid.

The test starts by filling the lower compartment with the aqueous xanthan gum solution, then the upper compartment with the particle-laden suspension. We open the outlet at the bottom of the lower compartment to initiate fluid flow, and use a high-resolution scale to continuously monitor the total discharge mass (OHAUS Valor 7000). Figure S1 in the supplementary materials illustrates the discharged mass as a function of time for a typical test; “falling head” boundary condition justifies the decrease in flow rate during the experiment, even in the absence of particles and clogging. While flow rate affects particle migration and clogging when *ρ*_*p*_ > *ρ*_*f*_ (gravity and inertial retardation^12^), changes in flow rate have a minor effect on clogging when particles are neutrally buoyant (*ρ*_*p *_*≈ ρ*_*f*_).

We also image the 3D granular dome that forms above the openings during dry granular flow (X-ray tomography, pixel resolution: 40–100 μm). The post-processing image analysis involves the “watershed” algorithm to separate and identify individual particles (Avizo, Thermo Fisher Scientific).

## Experimental results

Particles form a bridge when they arrive quasi-simultaneously at the orifice^11,32^. The probability that the needed number of particles arrive at the orifice in the correct configuration decreases rapidly with *d*_*o*_/*d* and increases with the initial particle volume fraction or solidity *Φ*_*i*_. Dry granular flow experiments have the highest inlet solidity *Φ*_*i*_ and set the upper bound for the constriction-to-particle size ratio *d*_*o*_/*d* that can experience clogging for a given flow volume.

### Dry granular flow

**Figure 3:**
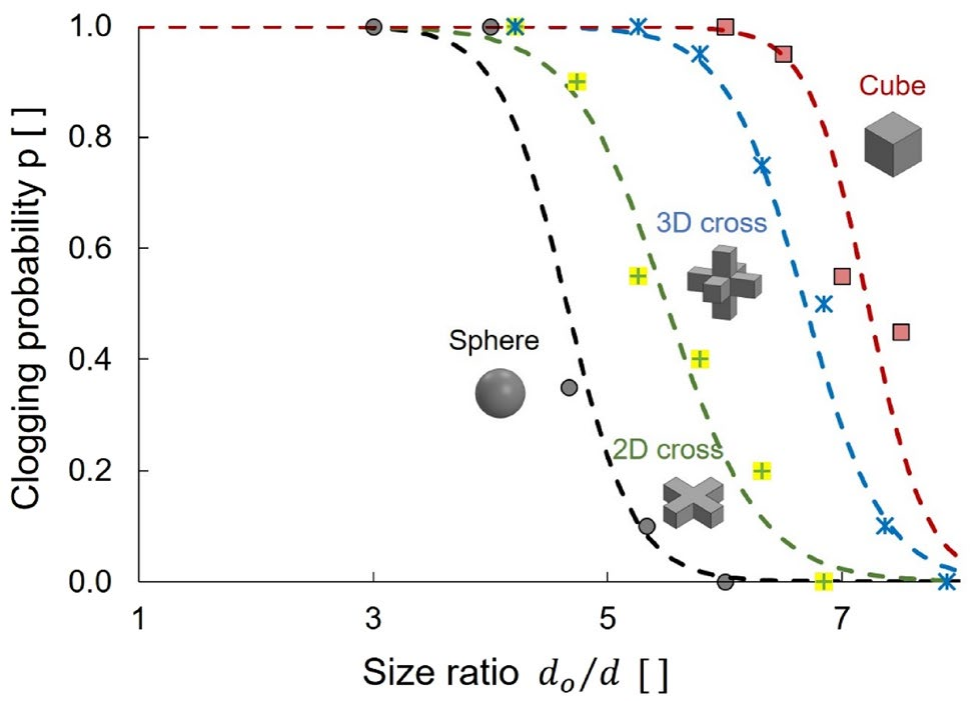
Dry granular flow: Clogging probability as a function of orifice-to-particle size ratio *d*_*o*_/*d* for the spheres, 2D crosses, 3D crosses and cubes (experimental results). The clogging probability is the ratio of the number of tests that clogged (within a maximum discharge of 4000 particles for spheres, 2D and 3D crosses, and 2000 particles for cubes) to the total number of tests (20 trials). Datapoints: 360 independent experimental realizations. Lines: logistic function (Eq. 2).

We define the clogging probability *p* during dry granular flow tests as the ratio of the number of tests that clog within a maximum discharge of 4000 particles (2000 particles in experiments with cubes), to the total number of tests. Figure 3 illustrates the clogging probability as a function of *d*_*o*_/*d* ratio for the different particle shapes. A logistic function fits experimental results (Fig. 3):

2 $$\it p=\frac{1}{1+\mathrm{exp}(-k\cdot {\left({d}_{o}/d-{(d}_{o}/d\right)}_{50})}$$

This equation has two fitting parameters: *k* is the logistic function growth rate, and (*d*_*o*_/*d*)_*50*_ is the size ratio with a clogging probability of *p* = 0.5. Cubes are the most prone to clogging for a given size ratio and form the largest granular bridges during dry granular flow with (*d*_*o*_/*d*)_*50*_ = 7.2, followed by the 3D crosses (*d*_*o*_/*d*)_*50*_ = 6.7, 2D crosses (*d*_*o*_/*d*)_*50*_ = 5.5 and spheres (*d*_*o*_/*d*)_*50*_ = 4.7.

**Figure 4:**
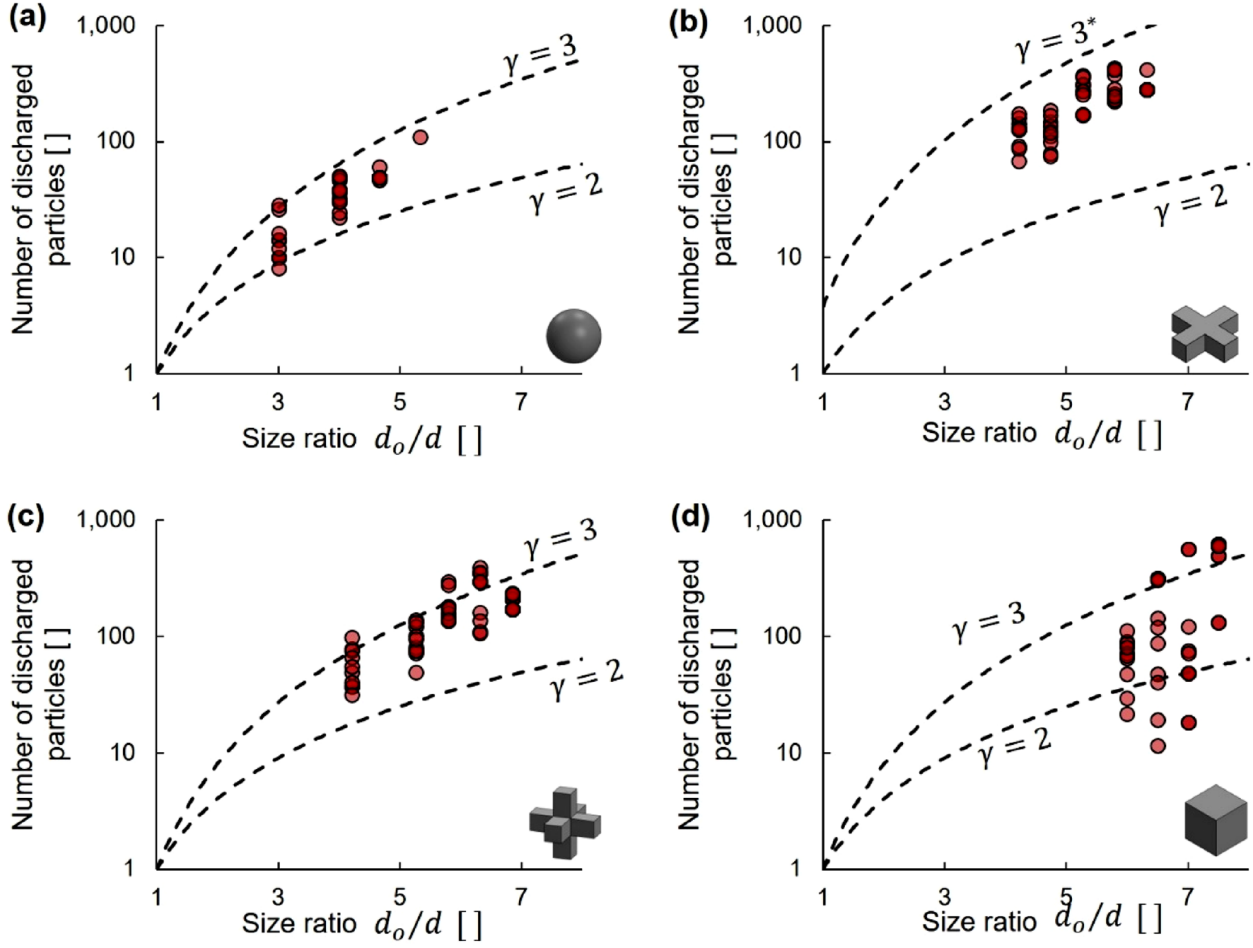
Dry granular flow: number of particles discharged before clogging as a function of the size ratio *d*_*o*_/*d.* Granular shapes: (**a**) spheres, (**b**) 2D cross, (**c**) 3D cross and (**d**) cubes. Datapoints: 134 independent experimental realizations. Lines: power function (Eq. 3). The γ-exponents selected to capture the lower (γ = 2) and upper (γ = 3) bounds. *The upper bound for the 2D cross data takes into consideration particle flatness (Eq. 4).

Figure 4 illustrates the number of particles discharged before clogging for the different shapes during dry granular flow. We fit the data in Fig. 4 with a power function:

3 $${N}_{d}={\left({d}_{o}/d\right)}^{\gamma }\, applies \,to\, {d}_{o}/d\ge 1$$

Granular domes either readily form soon after uncovering the orifice or the entire hopper empties. Thus, the particle discharge data in Fig. 4 reflects the structure of the granular dome that forms above the orifice immediately after releasing the plug. The one-particle discharge asymptote *N*_*d*_ = 1 for size ratio *d*_*o*_/*d *→ 1 implies that bridging forms instantaneously upon unplugging the orifice and that only one particle can fall if sitting immediately above the orifice.

The lower bound exponent γ = 2 represents a discharge of one layer of particles sitting on the orifice. The upper bound exponent γ = 3 represents the number of particles that occupy a volume $\propto$ *d*^3^ just above the orifice with a solidity similar to simple cubic packing. The 2D crosses are anisotropic, thus, the upper bound for these particles takes into consideration their thickness *d*_*t*_:

4 $${N}_{d}={\left({d}_{o}/d\right)}^{2}\left({d}_{o}/{d}_{t}\right)={\left({d}_{o}/d\right)}^{3}\left(d/{d}_{t}\right)$$

where *d*/*d*_*t*_ is a measure of particle flatness. Indeed, experimental results show that the particle discharge for the 2D crosses is higher than for the other shapes.

### Particle-laden fluid flow

The observed sequence of events leading to clogging at large *d*_*o*_/*d* ratios during particle-laden flow is similar for all shapes and test conditions. At the beginning, particles form unstable bridges that readily collapse (intermittent clogging). Increasingly, particles accumulate around and above the orifice increasing the local solidity *Φ*. After multiple bridging and destabilization events, a stable bridge forms and additional particles soon become trapped behind the bridge and contribute to its stability. Clearly, bridge formation at pore constrictions is a stochastic event when particles are in suspension.

**Figure 5:**
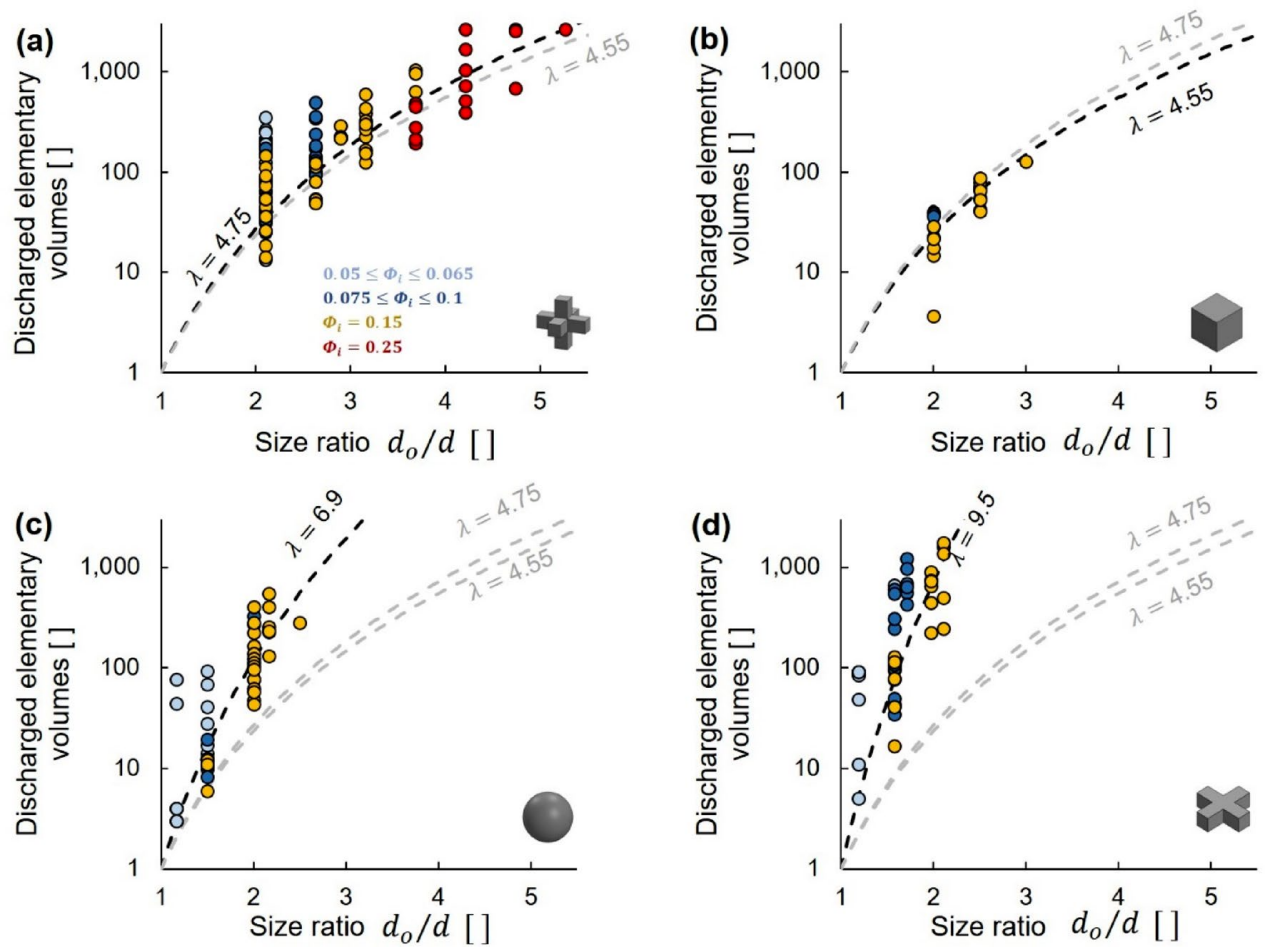
Particle-laden fluid flow: discharged elementary volumes before clogging as a function of orifice-to-particle size ratio *d*_*o*_/*d*. Granular shapes: (**a**) 3D crosses, (**b**) cubes, (**c**) spheres and (**d**) 2D crosses. Datapoints: 280 independent experimental realizations, colored by initial suspension solidity *Φ*_*i*_. Lines: power function (Eq. 5). The *λ*-exponent is a shape-related fitting parameter. Light gray lines added for relative reference among plot.

We repeat the tests for each shape, suspension solidity *Φ*_*i*_ and orifice-to-particle size ratio *d*_*o*_/*d* to obtain statistical trends for clogging. Let’s define the elementary volume *V*_*e*_ as the suspension volume associated with one particle (Note: *V*_*e*_ = *V*_*p*_/*Φ*_*i*_ where *V*_*p*_ is the volume of a particle). Then, the number of discharged elementary volumes is the total discharged volume *V*_*d*_ divided by *V*_*e*_. Figure 5 illustrates the number of discharged elementary volumes as a function of the size ratio *d*_*o*_/*d* for the different shapes. We fit the data in Fig. 5 with a power-law model to obtain quantitative parameters that enable us to compare the clogging tendencies of different shapes (see related analytical solution^11^):

5 $$\frac{{V}_{d}}{{V}_{e} }={\left({d}_{o}/d\right)}^{\lambda } \,applies\, to\, {d}_{o}/d\ge 1$$

where the shape-dependent *λ*-exponent decreases as particles favor clogging (Fig. 5). The high λ-exponents (λ = 4.55–9.5—Fig. 5) highlight the fast increase in expected discharge volume with orifice-to-particle size ratio *d*_*o*_/*d*. This response is a consequence of two concurrent trends as the number of particles required to form the bridge increases: (1) the probability that all needed particles will arrive simultaneously decreases and (2) the stability of larger bridges becomes increasingly more sensitive to the relative position of neighboring particles. Therefore, while clogging is theoretically possible for any *d*_*o*_/*d* size ratio, its likelihood becomes diminishingly small for large orifice sizes. The lower asymptote of the adopted power-law model reflects the fact that clogging is guaranteed when particles are larger than the orifice *d*_*o*_/*d* < 1; then, at most one elementary volume could drain before clogging (Note: the expected value is, *V*_*d*_/*V*_*e *_→ 0.5 for *d*_*o*_/*d* < 1).

**Figure 6:**
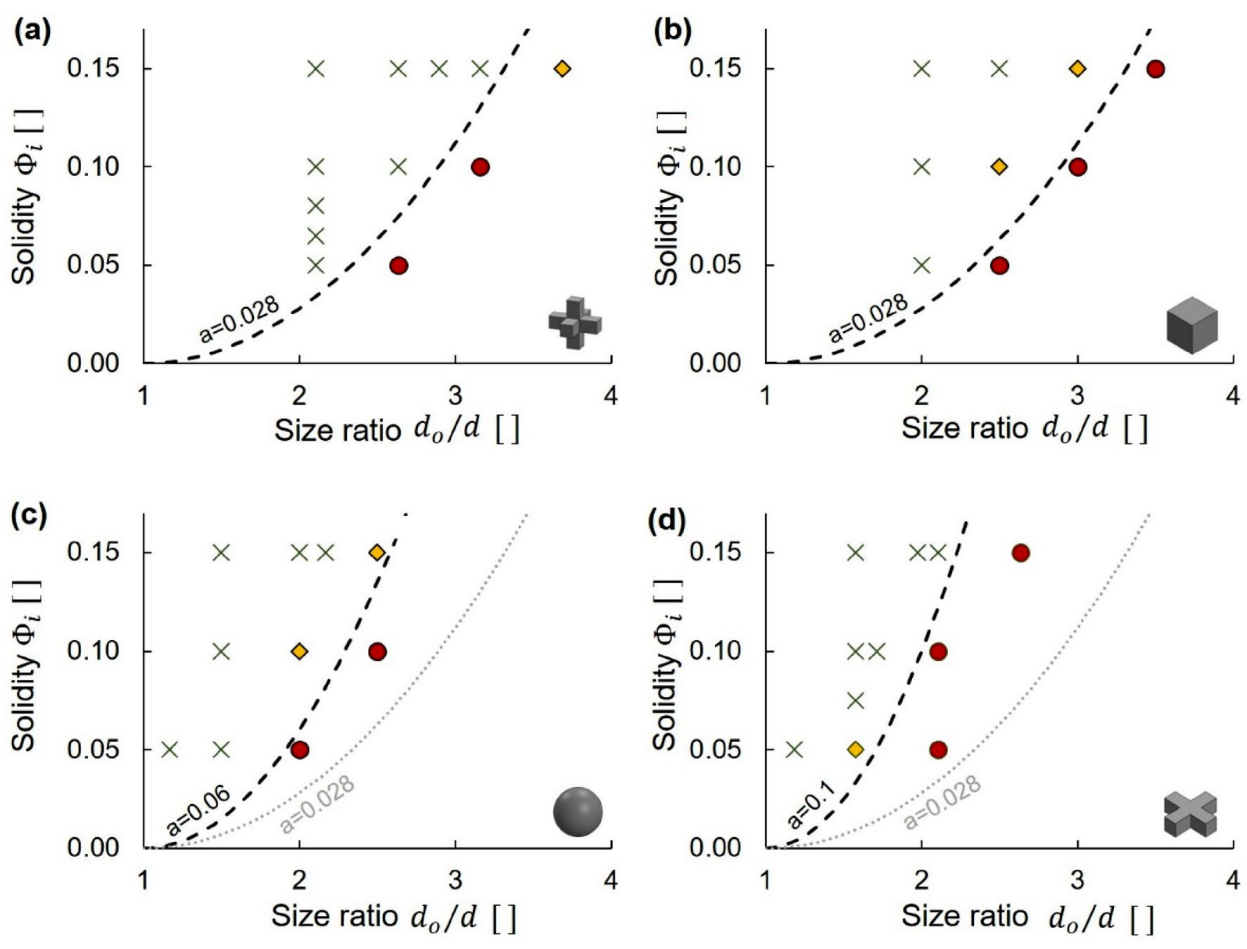
Particle-laden fluid flow: Clogging domains before a discharge suspension volume *V*_*s*_ < 800 ml (experimental results). Space defined by the initial suspension solidity *Φ*_*i*_ and the orifice-to-particle size ratio *d*_*o*_/*d*. Green: clogging probability *p* = 1. Red: *p* = 0. Yellow: 0 < *p* < 1. Lines: domain boundaries follow a second-order power equation $\phi = a{[\left({d}_{o}/d\right)-1]}^{2}$. The light gray lines added for mutual reference. Figure represents 280 independent experimental realizations.

We run complementary tests to define the “clog” versus “non-clog” domains in the *Φ*_*i*_-versus-*d*_*o*_/*d* space for a given total discharge suspension volume *V*_*s*_. Figure 6 summarizes the effect of particle shape on domain boundaries for a discharge suspension volume *V*_*s*_ < 800 ml. These results clearly show that the 3D crosses and cubes clog more readily for all solidities *Φ*_*i*_, followed by the spheres and 2D crosses; this trend is consistent with the fitted shape λ-exponent in Fig. 5. Domain boundaries superimposed on Fig. 6 correspond to $\phi = a{[\left({d}_{o}/d\right)-1]}^{2}$ where $a$ is a fitting parameter. Clogging is guaranteed when *d*_*o*_/*d* < 1 even for the minimum possible initial solidity of one particle in the available fluid volume, *Φ*_*i,min*_*.* As the initial fluid volume can be extended to any value, we adopt the asymptote *Φ*_*i,min*_ = 0 for *d*_*o*_/*d *→ 1.

Differences between domain boundaries for the cubes and 3D crosses are within the margin of error so we cannot conclude about their relative propensity to clog.

### Bridge formation

The geometrical analysis of bridges made of spherical particles relates the size ratio *d*_*o*_/*d*, the angle between two adjacent particles ζ, and the number of particles *n* in the bridge^33^:

6 $$\frac{{d}_{o}}{d}=\frac{\mathrm{sin}\left(\frac{n\pi -n\zeta }{2}\right)}{\mathrm{cos}\left(\frac{\zeta }{2}\right)}$$

For spherical particles to form a dome, a number *n* of particles must simultaneously arrive at the orifice with the correct inter-particle angle ζ. Spheres that arrive with an incorrect configuration will allow slippage and prevent dome formation. The limited inter-particle contact area and lack of interlocking hinders bridge formation by spherical particles.

Cube-cube interaction can sustain a torque and cubes do not roll freely^1,34^, so cubic particles may not slip past each other even when they arrive in slightly incorrect configurations. Face-to-face or face-to-edge interactions cause several cubic particles to move in clusters and behave like a single larger particle^22,27,34^.

Hindered particle rotation and interlocking prevail among 3D crosses. Two or more particles often “aggregate” during flow and reach the orifice as a larger cluster^26,27^. There is rapid clogging by simultaneous arrival at *d*_*o*_/*d* < 3 and intermittent clogging at *d*_*o*_/*d* > 3 and *Φ*_*i*_ = 0.25, indicative of bridge destabilization and particle accumulation. For comparison, intermittent clogging of spherical particles during particle-laden fluid flow has been observed for 3 < *d*_*o*_/*d* < 4^35,36^.

The flat 2D crosses orient their longest axes in the direction of flowlines across the orifice (similar observations for mica platelets^32^). As a result, a large number of the 2D crosses needs to arrive simultaneously at the orifice to form an arch^3^. Clearly, deviation from sphericity is needed in all three dimensions in order to favor bridge formation.

**Figure 7:**
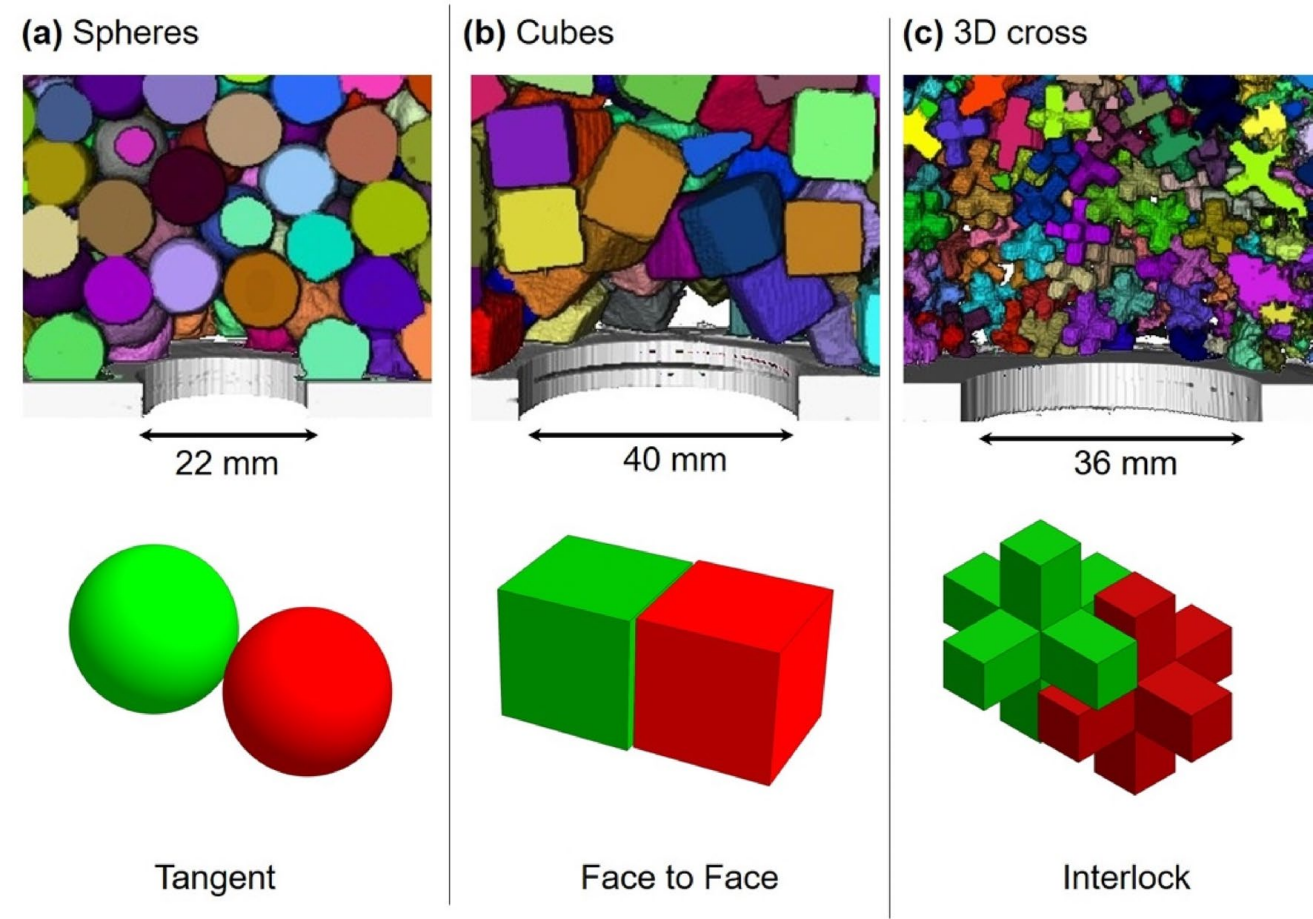
Bridge topology (experimental results). CT scans of bridges formed by (**a**) spheres, (**b**) cubes and (**c**) 3D crosses above the orifice during dry granular flow. Typical particle–particle interaction modes are shown below each scan.

X-ray tomographic images reveal the unique particle assemblages that form the dome-like structures for different particle shapes (Fig. 7), and underlying interparticle interactions: through a single point contact between spherical particles (Fig. 7a), face-to-face and face-to-edge contacts among cubic particles (Fig. 7b) and multiple contact points and particle interlocking among 3D crosses (Fig. 7c). The resulting cavity geometry reflects these interactions, ranging from regular domes made of spherical particles, a column-beam structure formed by cubes, and non-convex openings formed by interlocking crosses (CT images in Fig. S2).

## Analyses and discussion

### Dry granular flow versus particle-laden fluid flow

The initial packing density or solidity *Φ*_*i*_ of dry particles before hopper discharge differs with particle shape; in particular, the initial solidity of cubes (*Φ*_*i*_ = 0.83) is significantly higher than for other shapes *Φ*_*i*_ = 0.45 to 0.68 (Fig. 1). In addition, cubic particles align with the hopper walls, form ordered columnar structures during granular flow and effectively decrease the hopper cross-sectional area. Reduced effective hopper size and high packing density explain the high clogging probability of cubes during dry granular flow (Fig. 3).

Angle of repose measurements capture particle relative mobility and hint to the propensity of different shapes to clog (Fig. 1). Cubes do not follow the general trend, as clogging by cubic particles benefits from their inherent tendency to form ordered columnar structures that can resist bending (i.e., interparticle torque). Sphericity *ψ* and initial solidity *Φ*_*i*_ do not capture the complex particle interactions during flow, including particle ordering, interlocking and alignment, and cannot fully predict the relative clogging tendencies of different shapes.

Dense particles experience inertial retardation when the fluid streamlines bend around constrictions or gravitational retardation due to self-weight^13,15^. Our particle-laden fluid flow experiments were designed with neutrally buoyant particles *ρ*_*p *_*≈ ρ*_*f*_ to minimize inertial effects. Yet, we observed “kinetic retardation”, i.e., the gradual local accumulation of particles during intermittent clogging prior to stable bridge formation. In all cases, granular retardation results in a gradual increase in the local particle volume fraction or solidity above the constriction, which increases the probability of clogging.

Finally, we expect the force distribution within the granular pack that forms above the orifice to be strongly influenced by fluid drag and to change as particles accumulate against the bridge. Bridges with limited stability fail to take the load, thus, particle-laden fluid flow often experiences intermittent clogging.

### Discharge distribution

Previous research results gathered with spherical particles show that the probability of clogging is constant and time independent during dry granular flow and particle-laden fluid flow, and that the discharge mass before clogging for a specific size ratio *d*_*o*_/*d* follows an exponential distribution^32,35^. However, these observations do not hold for non-spherical particles^37,38^.

**Figure 8:**
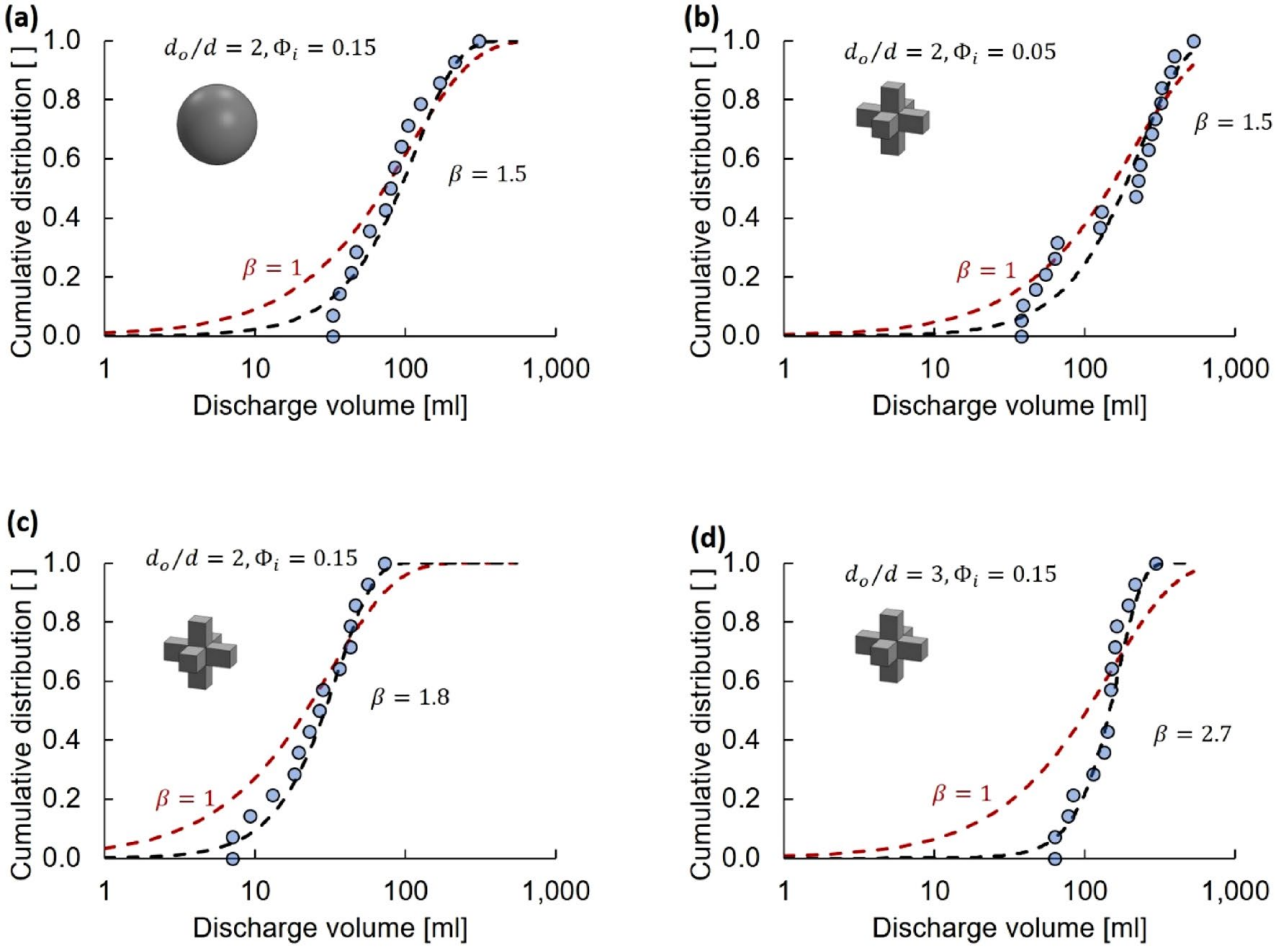
Discharge volume: statistical analysis (experimental results). Cumulative distribution of the discharge volume before clogging *V*_*d*_ for (**a**) spheres and (**b**–**d**) 3D crosses at different initial suspension solidity *Φ*_*i*_ and size ratios *d*_*o*_/*d*. Datapoints: experimental cumulative distribution based on 14 or more experiments. Red dashed line: exponential distribution function (*β* = 1). Black dashed line: Weibull distribution function (Eq. 7). *β*: Weibull shape parameter.

We test these observations for particle-laden fluid flow by analyzing the distribution of discharge volume before clogging. Figure 8 illustrates the cumulative distributions obtained from the measured discharge volume before clogging for spheres and 3D crosses. Each cumulative distribution includes data from more than 14 tests. We fit the discharge volume before clogging with the two-parameter Weibull distribution function^39^:

7 $$F\left({V}_{d}\right)={\int }_{0}^{{V}_{d}}f\left({V}_{d}\right)d{V}_{d}=1-{e}^{{\left(-\frac{{V}_{d}}{\alpha }\right)}^{\beta }}$$

where *α* and *β* define the distribution. The shape parameter is *β* > 1 when the event rate increases with discharge volume *V*_*d*_. The Weibull distribution reduces to the exponential distribution when *β* = 1.

The Weibull distribution fits the experimental data with exponents close to *β* = 1 for low size ratios *d*_*o*_/*d* (spherical particles: *d*_*o*_/*d* = 2, *Φ*_*i*_ = 0.15, *β* = 1.5; 3D crosses: *d*_*o*_/*d* = 2, *Φ*_*i*_ = 0.05, *β* = 1.5). However, the Weibull shape parameter increases at large *d*_*o*_/*d* ratios (3D crosses: *d*_*o*_/*d* = 2, *Φ*_*i*_ = 0.15, *β* = 1.8 and *d*_*o*_/*d* = 3, *Φ*_*i*_ = 0.15, *β* = 2.7). These results suggest that the clogging of small orifices *d*_*o*_/*d* < 3 is a Poisson process, however, the clogging probability becomes history-dependent for large *d*_*o*_/*d* ratios and large solidities *Φ*_*i*_ where particle–particle interactions and interlocking gain significance. Kinetic retardation and local particle accumulation near the orifice enhance particle interactions and increases $\beta$.

## Conclusions

We investigated the discharge and clogging behavior of spherical, elongated, faceted, and non-convex particles during both dry-granular and particle-laden fluid flow. Results highlight the role of particle shape on the average discharge volume before clogging, which also depends on the orifice to particle size ratio *d*_*o*_/*d* and initial solidity *Φ*_*i*_.

Particle shape defines particle-to-particle interaction and relative mobility. Cubes and 3D crosses are the most prone to clogging. The superior clogging performance of the 3D crosses results from their ability to interlock. Face-to-face contacts among cubes can resist torque and enhance the clogging probability. Consequently, particle-to-particle interactions define the geometry of clogging domes: column-beam geometry for cubes, non-convex cavities for interlocking crosses, and regular domes formed by spherical particles.

We tested neutrally buoyant particles. Still, intermittent bridge formation and destabilization events contribute to the gradual increase in local solidity near the orifice. Therefore, kinetic retardation and inertial retardation promote clogging.

The cumulative distribution of discharge volume before clogging follows a Poisson distribution for spherical particles, which indicates that clogging is a random process. However, non-spherical particles exhibit a Weibull cumulative distribution for volume discharge prior to clogging at large *d*_*o*_/*d* ratios and solidity *Φ*_*i*_. Clogging by non-spherical particles is history-dependent due to particle–particle interactions and retardation.

We adopted functions with physically justifiable asymptotic trends and with a minimum number of parameters (Ockham’s criterion) to compare the clogging tendencies. While the model parameters relate to particle shape, index properties such as angle of repose, sphericity and initial solidity do not capture the complex particle interactions during granular flow and cannot fully predict the relative clogging tendencies of different shapes.

## Supplementary information

Supplementary information.

## Abbreviations

$A_{p} \,\left[ {{\text{m}}^{2} } \right]$: Particle surface area
$$d_{o} \,\left[ {\text{m}} \right]$$: Orifice size
$$d \,\left[ {\text{m}} \right]$$: Particle size
$\left( {d_{o} /d} \right)_{50} \left[{}\right]$: Characteristic orifice-to-particle size ratio with clogging probability *p* = 0.5
$k \left[ {} \right]$: Logistic function growth rate
$n \left[ \right]$: Number of particles in a bridge
$N_{d} \left[ \right]$: Number of discharged particles before clogging
$$p \left[ {} \right]$$: Clogging probability
$V_{e} \,\left[ {{\text{ml}}} \right]$: Elementary fluid volume
$V_{d} \,\left[ {{\text{ml}}} \right]$: Discharged volume before clogging
$$V_{p} \, \left[ {{\text{m}}^{3} } \right]$$: Particle volume
$V_{s} \,\left[ {{\text{ml}}} \right]$: Discharged suspension volume
$\alpha \left[ \right]$: Weibull scale parameter
$\beta \left[ \right]$: Weibull shape parameter
$\varPhi \left[ \right]$: Solidity = (volume of particles)/(total volume)
$\lambda \left[ \right]$: Fitted shape coefficient
$\rho_{p} \left[ {{\text{g}}/{\text{cm}}^{3} } \right]$: Particle mass density
$\rho_{f} \left[ {{\text{g}}/{\text{cm}}^{3} } \right]$: Fluid mass density
$\zeta \left[^\circ \right]$: Inter-particle angle
$$\psi \left[ \right]$$: Particle sphericity
