# Supplementary material for: The effect of particle shape on discharge and clogging
Source: Sci Rep. 2021 Feb 8;11:3309. doi: 10.1038/s41598-021-82744-w (PMC7870973; doi:10.1038/s41598-021-82744-w)
Supplement: Supplementary file 1 — Supplementary information. [file 41598_2021_82744_MOESM1_ESM.docx]

**Supplementary information**

**The Effect of Particle Shape on Discharge and Clogging**

Ahmed Hafez^1^, Qi Liu^1^, Thomas Finkbeiner^1^, Raed A. Alouhali^2^, Timothy E. Moellendick^2^, J. Carlos Santamarina^1*^

**Affiliation**: ^1^ Earth Science and Engineering, KAUST, Thuwal 23955-6900, Saudi Arabia.

^2^ Saudi Aramco, Dhahran 31 311, Saudi Arabia.

**Corresponding author ***: J. Carlos Santamarina, carlos.santamarina@kaust.edu.sa

Fig. S1: Cumulative discharged fluid mass as a function of time in a typical particle-laden flow experiment.


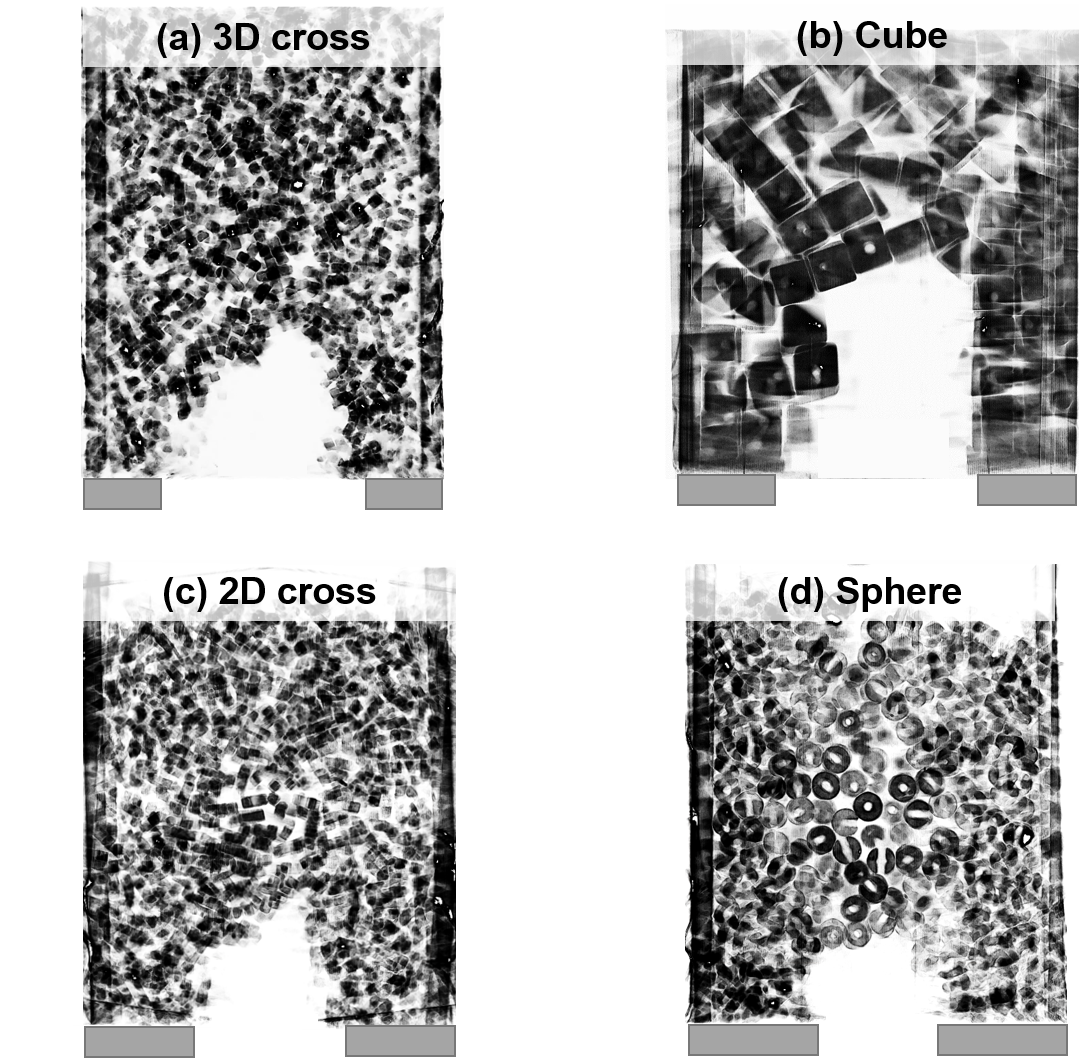


Fig. S2: Cavities formed during dry granular flow tests. CT slice. (a) 3D crosses (d_o_/d=5.8), (b) cubes (d_o_/d=5), (c) 2D crosses (d_o_/d=4.2), and (d) spheres (d_o_/d=4). The lower rectangles define the orifice position and size. Particle shape defines particle-to-particle interaction and relative mobility. The superior clogging performance of the 3D crosses results from their ability to interlock. Face-to-face contacts among cubes can resist torque and enhance the clogging probability. Such particle-to-particle interactions define the geometry of clogging domes: column-beam geometry for cubes, non-convex domes formed by interlocking crosses, and regular domes formed by spherical particles.
